# Arteriovenous Fistula: The Case of a Rare Complication after Minimal Percutaneous Nephrostomy and Brief Review

**DOI:** 10.3390/diagnostics14111121

**Published:** 2024-05-28

**Authors:** Răzvan Alexandru Dănău, Răzvan-Cosmin Petca, Traian Vasile Constantin, Aida Petca, Gabriel Predoiu, Viorel Jinga

**Affiliations:** 1 Department of Urology, “Carol Davila” University of Medicine and Pharmacy, 8 Eroii Sanitari Blvd., 050474 Bucharest, Romania; razvan.danau@umfcd.ro (R.A.D.); traian.constantin@umfcd.ro (T.V.C.); gabriel.predoiu@umfcd.ro (G.P.); viorel.jinga@umfcd.ro (V.J.); 2 Department of Urology, “Prof. Dr. Th. Burghele” Clinical Hospital, 20 Panduri str., 050659 Bucharest, Romania; 3 Department of Obstetrics and Gynecology, “Carol Davila” University of Medicine and Pharmacy, 8 Eroii Sanitari Blvd., 050474 Bucharest, Romania; aida.petca@umfcd.ro; 4 Department of Obstetrics and Gynecology, Elias University Emergency Hospital, 17 Mărăști Blvd., 050474 Bucharest, Romania; 5 Medical Sciences Section, Academy of Romanian Scientists, 050085 Bucharest, Romania

**Keywords:** arteriovenous fistula, percutaneous nephrostomy, embolization

## Abstract

Percutaneous renal surgery, although much less invasive than other procedures, is subject to several complications, which can occur at any time during the course of treatment, starting from the performance of the minimal nephrostomy procedure. We present an extremely rare vascular complication of percutaneous nephrostomy represented by arteriovenous fistula that occurred in a 24-year-old patient known to have right ureteropelvic junction obstruction operated with the absence of double-J catheter permeability and grade II-III hydronephrosis for which minimal percutaneous nephrostomy was urgently fitted. The arteriovenous fistula was resolved by supraselective artery embolization.

## 1. Introduction

Congenital obstructive abnormalities of the upper ureter and pelvis, although common, are underdiagnosed, sometimes being asymptomatic, in 10–15% of cases encountered as a random finding. The absence of specific symptomatology is due to the fact that the pelvis acts as an “expansion chamber”; imaging explorations are usually performed in the context of recurrent episodes of urinary tract infection or are used for the differential diagnosis of other painful conditions.

Ureteropelvic junction (UPJ) obstruction often accompanies an inferior polar vessel, requiring knowledge of the percutaneous access technique. The abnormalities may be isolated or combined, sometimes bilateral. Following the disruption of urinary drainage, hydronephrosis occurs. Diagnosis, taking as a starting point the symptoms of pain, urinary infection, and hematuria, is based on investigations such as ultrasonography, urography, computed tomography, retrograde ureteropyelography in selected cases, and renal scintigraphy [1,2]. Urinary drainage, both by percutaneous nephrostomy and internal double-J catheters, is the first choice, especially in cases associated with septic complications.

Percutaneous nephrostomy, a procedure established in the early 20th century, allows for the drainage of the renal collecting system and is performed under ultrasound, computer tomography (CT), or rotational fluoroscopy guidance. Percutaneous nephrostomy can be performed independently or combined with endoscopic or surgical techniques on native and transplanted kidneys [1,2]. The main indications for percutaneous catheterization are urinary drainage—to preserve renal function and prevent septic complications; calculi extraction when the nephrostomy tract is subsequently used for percutaneous nephrolithotomy (PCNL); and, in selected cases, the insertion of double-J catheters via anterograde access or insertion of medication [3,4,5,6]. In addition to its therapeutic role, it also has a diagnostic role, in which it is used to inject contrast medium to visualize the renal collecting system.

Thus, percutaneous nephrostomy allows urinary drainage, whether there is an intrinsic or extrinsic urinary obstruction (calculi, tumors, iatrogenic) with or without infection, representing an effective solution in 84 to 99% of cases [2,3]. Sepsis with a urinary entry point associated with obstructive uropathy is a severe complication with a high mortality rate.

It is accompanied by several contraindications, the most important of which are severe coagulopathies (imposed by the risk of bleeding induced by puncture and subsequent dilatation of the tract through the renal parenchyma) or terminal diseases. In 2016, the percutaneous nephrostomy protocol was published [3,7,8]. Additional precautions are also required in patients with severe thrombocytopenia, prolonged thromboplastin or prothrombin times, in those with severe pulmonary impairment when procubitus is not recommended, or in the case of musculoskeletal malformations or retrorenal colon.

The complications of this method are those associated with any minimally invasive surgical procedure: vascular injury, damage to nearby organs, and infection [9,10,11,12,13,14,15,16]. The Society of Cardiovascular and Interventional Radiology and the American College of Radiology have shown that percutaneous nephrostomy is associated with less than 4% major bleeding or septic shock [17]. Vascular injury can occur both at the time of puncture and minimal tract dilation by intercepting the vessels and during catheter movement. Symptomatology is abrupt, caused by hyperdistension of the pyelocaliceal system through clot accumulation causing colicky pain and hemorrhage that may be intrarenal or extrarenal and requires, for diagnostic certainty, either evidence of vascular lesion (aneurysm or arteriovenous fistula) by Doppler ultrasonography or fluoroscopic exploration (arteriography) [1,9,10,15,18,19].

In a study that included 318 patients undergoing percutaneous nephrostomy, Lewis and Patel [17] found vascular complications in 0.6% of the cases studied, concluding that percutaneous nephrostomy is a safe procedure with insignificant complications. The arteriovenous fistula may remit spontaneously due to the buffering effect of the surrounding tissue, which requires careful monitoring. It is also the first therapeutic approach after removing the external urinary drain with the occurrence of hematuria. If bleeding increases, embolization via an arterial catheter or surgery is required. Another complication common in patients with percutaneous nephrostomy is infection or sepsis. There are different results in the literature regarding infection: 2.5–19% [18]. Regarding the risk of infection, the protocol shows the need to monitor these patients and prophylactic antibiotic therapy in those at high risk. There may also be rare complications such as renal–digestive fistula or urinothorax [20,21].

This paper aims to describe an extremely rare vascular complication of percutaneous nephrostomy represented by arteriovenous fistula, resolved in a conservative manner by supraselective embolization.

## 2. Case Report

**Figure 1:**
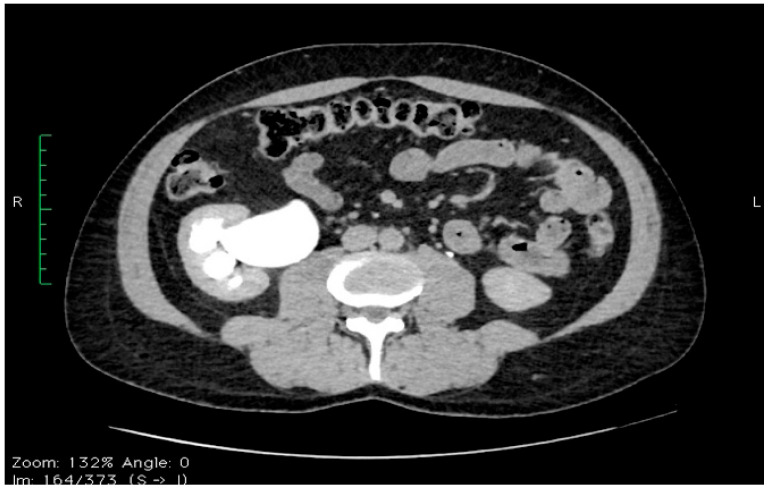
Axial CT section—the preoperative appearance of right UPJ obstruction with extrasinusal pelvis and distension of the calyceal cups.

A 24-year-old patient came to the urology service for intermittent right lumbar pain with minimal anteroinferior dull radiation with spontaneous onset and remission. Suspicion of UPJ obstruction was raised, subsequently confirmed by CT examination and renal scintigraphy—Figure 1.

Right Hynes–Anderson pyeloplasty was performed via laparoscopic access with protection of the anastomosis site with a double-J 6 Ch catheter. The postoperative course was uncomplicated, the lateral anastomotic drainage was suppressed after 48 h, and the patient was discharged on the 4th day without perirenal collections and pyelocaliceal distention. Additionally, the positioning of the double-J catheter with the superior volute at the pelvic level was verified fluoroscopically.

After 30 days, the patient presented to the emergency department with colicky right lumbar pain, a febrile state that started 48 h before, and significant leukocytosis (WBC 19,800/µL). An ultrasonography was performed at first, which revealed the presence of a double-J catheter at the pelvic level, but with grade II hydronephrosis and inhomogeneous content in the renal cavities. 

Minimal percutaneous echo-guided nephrostomy was installed, through which pyuria was exteriorized, and broad-spectrum antibiotic treatment—carbapenems—was initiated. Algic symptoms, febrile syndrome, and leukocytosis were remitted within 48 h of treatment onset. Forty-eight hours after the insertion of the external drain, the right double-J catheter was removed, and right semirigid hypopressure ureteroscopy was performed (under nephrostomy protection) showing edema at the level of pelvis–ureteral anastomosis, wide caliber, followed by double-J catheter replacement. The nephrostomy tube was clamped, and further ultrasound exploration was performed, confirming permeability and the absence of distention of the pyelocaliceal system. The minimal nephrostomy was removed, and a compressive dressing was applied.

**Figure 2:**
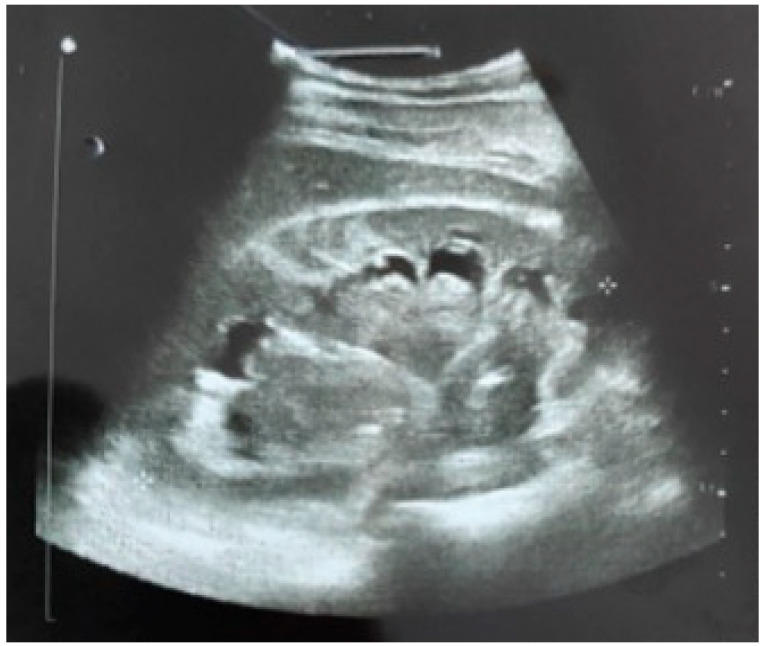
Ultrasonographic aspect—grade II-III hydronephrosis with clots in the collecting system. The double-J catheter is visualized in the pelvis.

Two hours after the removal of the external urinary prosthesis (it should be noted that at the time of removal, pulsatile bleeding was observed at the level of the tract, for which a compressive dressing was applied), the patient developed sudden colicky pain and significant hematuria. Ultrasonography showed grade II-III hydronephrosis with clots in all calyceal groups—Figure 2. The association of color Doppler examination raised the suspicion of an arteriovenous fistula at the level of the lower renal pole on the nephrostomy tract.

**Figure 3:**
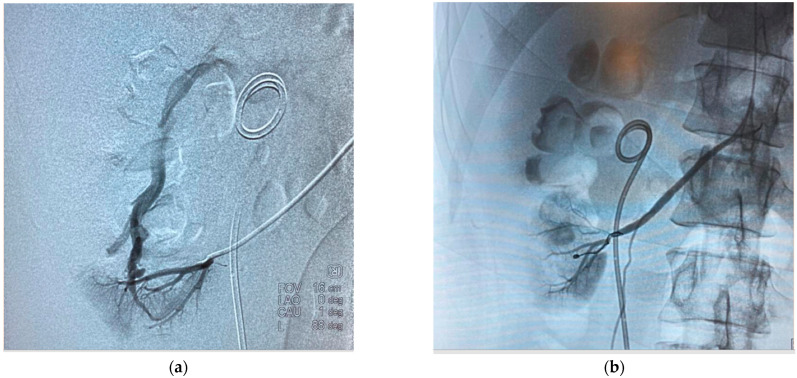
Fluoroscopic aspects: (**a**) confirmation of the presence of arteriovenous fistula; (**b**) appearance after supraselective embolization.

Given the sudden evolution, an emergency arteriogram was performed, confirming the presence of vascular complications, and was subsequently embolized supraselectively—Figure 3. The technique used involves puncture under local anesthesia of the right femoral artery in selected cases or, more commonly, the left brachial artery using the Sledinger technique and a 6F endovascular sheath, followed by abdominal aortography on a 0.0035-inch guide (Terumo, Osaka, Japan), with the injection of contrast medium allowing both renal arterial trunks to be highlighted. In the case of lesions in the vicinity of the segmental artery, 4F catheterization and embolization probes are used. Microcatheters are utilized for the subsegmental branches, which are inserted up to the area of the lesion, and then occlusive material is inserted. The intervention is considered successful when complete occlusion or total closure of bleeding is achieved on control angiography.

## 3. Discussion

Percutaneous nephrostomy is an effective method of external urinary bypass, with the percutaneous approach being successfully performed in 84–99% of cases. The main difficulty is imposed by the absence of distention of the pyelocaliceal system [22]. In addition to the therapeutic role of evacuating the collecting system (obstructions, fistulas, tumors, perirenal collections), it also plays a diagnostic role by highlighting obstructions by anterograde injection of contrast medium.

Complications of percutaneous nephrostomy have been classified into two broad categories, minor and major, which occur in 10% of cases together [23]. Of particular note is the low incidence of acquired arteriovenous fistulas requiring active treatment (embolization or nephrectomy), present, according to Pabon-Ramos W. M. et al., at an occurrence rate of between 0.1 and 1% [3]. Other major complications with an occurrence rate of less than 1% are pleural interception with pneumothorax, hydrothorax, or hemothorax and perforation of the colon at the puncture time [3].

Percutaneous surgery has become the “gold standard” for the minimally invasive treatment of various urological conditions. Most of the time, vascular complications are self-limited, transient, asymptomatic, or with minimal symptoms. A meta-analysis by Galoucii et al., which included a retrospective evaluation of 1000 patients who experienced vascular complications after percutaneous access, concluded that conservative treatment (repositioning of the nephrostomy tube, blood transfusions) is sufficient to control postoperative bleeding, with active treatment—embolization—being required in 0.03% of cases [24]. Of particular importance is that all procedures used a 24 F dilatation tract, performed through the inferior renal pole on the posterior axillary line. Under these conditions, significant intra- and postoperative bleeding was encountered in up to 10% of cases but with a transfusion being required in less than 3% of cases. The rate of occurrence of bleeding was directly proportional to the type of pathology (most cases were represented by urinary lithiasis followed by congenital UPJ stenosis and tumor pathology in selected cases) and the presence or absence of infection. The fitting of percutaneous nephrostomy as an emergency urological maneuver was not included [24]. 

Moreover, the data available in the literature on the risks of vascular interception for this treatment modality are extremely limited. A study by Lee et al. classified the presence of complications of this procedure according to the experience of the interventional surgeon or radiologist, concluding that although the less experienced had a higher rate of complications, they were minor and did not require active treatment [25]. For example, AbdelAziz reported in 2022 the therapeutic success of a nephrostomy tube placed for hemostatic purposes after percutaneous treatment of renal lithiasis (PCNL) with the tip migrated to the IVC, which did not require open repair by the vascular surgery team [7]. There was also evidence of an inferior polar arteriovenous fistula in the same patient, which required selective arterial embolization. It is again noted that these complications arose from a dilated tract, expanded to allow access for instrumentation. Other accidents in nephrostomy tube placement after prior dilation of the puncture tract included pleural interception with urinothorax, for which a pleural drain had to be fitted. Reducing the working tract by mini-PCNL development was assessed regarding vascular risk. 

Another extremely rare complication was reported by Herlambarg M.S. et al., which involved the development of a digestive fistula in the treatment of left coraliform lithiasis. As a first step, a minimal left nephrostomy was fitted. Anterograde injection of contrast medium revealed a continuity solution with the duodenum necessitating exploratory laparotomy, which confirmed the presence of the fistula, necessitating fistulectomy, closure of the gap, pyloromyotomy, and gastrojejunoanastomosis [20].

In the literature, data on vascular complications caused by minimal percutaneous nephrostomy insertion are extremely limited [3,24,25]—Table 1. Multiple international databases such as PubMed and Science Direct have been searched, and so far, from the information obtained, this represents the first case of AV fistula occurrence after this therapeutic maneuver that had a fulminant evolution and required selective arterial embolization.

In our case, although technically performed correctly, percutaneous nephrostomy insertion was followed by the development of an arteriovenous fistula with loud symptomatology that required active intervention with supraselective embolization. 

Jinga et al. stressed the importance of supraselective transarterial embolization in urological patients after PCNL. Thus, they concluded, after evaluating a group of 2097 patients, that the incidence of arteriovenous fistula and pseudoaneurysms post-PCNL was 1.05%, with the main risk factors identified being perinephritis, pyelonephritis, and multiple dilatation tracts, especially those located in the upper pole [26].

The development and implementation of “nephron-sparing” techniques, facilitated by introducing robotic-assisted procedures, has made it possible to address an increasing number of pathologies through a minimally invasive approach. In addition to the well-known benefits of robotic-assisted interventions, several complications specific to this approach have been described. Tufano et al. presented the case of an arteriovenous fistula secondary to robotically assisted partial nephrectomy in a patient without comorbidities, whose nephrorrhaphy was performed in two layers. The particularity of the case consisted of the absence of symptoms and hemodynamic changes, with the AVF being identified at one-year postoperation; the therapeutic option chosen in this case was the superselective embolization of the renal artery. Additionally, the authors describe, based on the literature data, a similar incidence of AVF occurrence at a rate of 1% after open surgery and 1.96% after a minimally invasive approach [27].

Renal parenchymal injury and renal vascular interception are considered to be the leading causes of this complication. Therefore, the endoscopic approach, which exclusively involves the urinary tract, is considered safe from vascular injury, with preoperative complications being classified as grades I and II according to the Clavien classification. Wan-Zhang Liu et al. described the occurrence of FAV in retrograde ureteroscopy for uncomplicated lithiasis pathology in a patient with distal ureteral lithiasis. Initially, the persistence of bleeding due to the double J was hypothesized but later refuted by imaging exploration (CT), which was requested due to a marked decrease in hemoglobin levels. The imaging assessment showed AVF, and the therapeutic option, in this case, was also represented by angiography and supraselective embolization. Although it is more frequently associated with the development of subcapsular hematoma than AVF, the risk factor was considered intrarenal hyperpressure [28]. According to the literature, in both flexible and semirigid ureteroscopy, the main risk factors identified are intrarenal hyperpressure associated with patients with arterial hypertension, injuries caused by the nitinol guidewire or ureteral access sheath, the development of subcapsular hematoma, and iatrogenic intraoperative injuries resulting from laser fragmentation [29].

The treatment of renal pathology, in its entirety, benefits from multiple approach options through open, robotic-assisted, or laparoscopic surgery, minimally invasive and endoscopic techniques, all with two major objectives: preserving renal function and reducing the risk of hemorrhage. Several risk factors have been identified as having significant value in the development of hemorrhagic complications. Thus, the presence of diabetes mellitus and renal anomalies have been implicated as positive predictive factors for the occurrence of post-interventional AVF [30]. Additionally, in the same study, a history of PCNL, ESWL, arterial hypertension, gender, age, presence of coagulopathies, and volume of the lithiasis were evaluated as variables in the development of AVF, but no statistically significant causative relationship was established. Another study, which evaluated 1400 patients, obtained similar results, with the main risk factor being the presence of fusion or rotation anomalies of the kidneys. In addition, a higher rate of hemorrhagic complications was observed in the case of staghorn lithiasis [31].

It is important to differentiate AVF from renal pseudoaneurysm (PA), even though both benefit from the same therapeutic approach and present with the same symptoms. AVF involves the interception of an arterial segment and a venous segment, whereas PA occurs secondary to arterial lumen lesions. Thus, AVF is more frequently encountered in procedures involving the renal hilum, while PA occurs more commonly in the renal parenchyma. In both situations, early diagnosis is essential, and the use of CT and angiography in case of persistent hematuria is mandatory, regardless of the type and surgical technique used, even though the incidence of this pathology is significantly reduced in the case of minimally invasive techniques, with a rate below 2%.

## 4. Conclusions

Percutaneous nephrostomy is a safe method of external urinary drainage, especially in patients with on-site internal urinary drainage, septic manifestations, and distention of the collecting system. Although it has an extremely low complication rate, the patient must be monitored both at insertion and removal to recognize complications early, with therapeutic maneuvers instituted early. Due to the development of interventional imaging techniques, nephrectomy for treating acquired arteriovenous fistulas has become more of a historical value.

## Figures and Tables

**Table 1:** Major complications after percutaneous nephrostomy [3,18,23,24,25].

Major Complications	Percentage (%)
Septic complications	1–10
Vascular complications requiring transfusions	
Simple percutaneous nephrostomy	1–4
In association with PCNL	12–14
Vascular injury requiring embolization or nephrectomy	0.1–1
Intestinal perforation	0.2–0.5
Pleural perforation	
Simple percutaneous nephrostomy	0.1–0.6
In association with PCNL	8.7–12
Complications leading to transfer to ICU, emergency surgery, or prolonged hospitalization	1–7

## Data Availability

Data are contained within the article.
